# Post-operative critical care management of patients undergoing cytoreductive surgery and heated intraperitoneal chemotherapy (HIPEC)

**DOI:** 10.1186/1477-7819-9-169

**Published:** 2011-12-19

**Authors:** Timothy J Cooksley, Philip Haji-Michael

**Affiliations:** 1 Department of Critical Care, The Christie, Manchester, M20 4BX, England

**Keywords:** Critical Care, Pseudomyxoma peritonei, HIPEC, Cytoreductive surgery

## Abstract

**Background:**

Cytoreductive surgery (CRS) and Heated Intraperitoneal Chemotherapy (HIPEC) results in a number of physiological changes with effects on the cardiovascular system, oxygen consumption and coagulation. The Critical Care interventions required by this cohort of patients have not yet been quantified.

**Methods:**

This retrospective audit examines the experience of a Specialist Tertiary Centre in England over an 18 month period (January 2009-June 2010) during which 69 patients underwent CRS and HIPEC. All patients were extubated in the operating theatre and transferred to the Critical Care Unit (CCU) for initial post-operative management.

**Results:**

Patients needed to remain on the CCU for 2.4 days (0.8-7.8). There were no 30 day mortalities. The majority of patients (70.1%) did not require post-operative organ support. 2 patients who developed pneumonia post-operatively required respiratory support. 18 (26.1%) patients required vasopressor support with norepinephrine with a mean duration of 13.94 hours (5-51 hours) and mean dose of 0.04 mcg/kg/min. Post-operative coagulopathy peaked at 24 hours. A significant drop in serum albumin was observed.

**Conclusion:**

The degree of organ support required post-operatively is minimal. Early extubation is efficacious with the aid of epidural analgesia. Critical Care monitoring for 48 hours is desirable in view of the post-operative challenges.

## Background

Pseudomyxoma peritonei is a rare epithelial neoplasm, characterized by progressive accumulation of peritoneal mucinous tumour deposition, usually originating from the appendix. In Western populations there is an estimated incidence of 1-2 per million a year [1].

Treatment of this condition with combined cytoreductive surgery (CRS) and hyperthermic intrapertioneal chemotherapy (HIPEC) has been shown to improve both patient survival and quality of life [2,3]. In this technique, the chemotherapy agent is typically perfused within the abdominal cavity for 90 minutes at a temperature of 42°C achieving high peritoneal concentrations with limited systemic absorption [4].

CRS and HIPEC has been depicted in some papers as a high risk procedure with high levels of morbidity (22-39%) [5], mortality (5%) [6] and prolonged hospital stays (up to an average of 29 days)[7]. The most common complications include anastomatic leaks, intra-abdominal sepsis, pancreatitis, intestinal fistula, renal failure and haematological toxicity. Furthermore, some Oncologists that it is the aggressive cytoreductive surgery alone that contributes to improved outcomes and that HIPEC may not have an impact on survival and simply adds unnecessary toxicity [5]. Factors that have been shown to significantly improve outcome include the peritoneal index (reflecting the disease burden) and the center in which the procedure is performed (those with > 7 years experience performing better) [8].

This complex procedure results in a number of physiological changes with effects on the cardiovascular system, oxygen consumption and coagulation [9-11]. Care of these patients presents a significant challenge to Anaesthetists and Critical Care Physicians. The Critical Care interventions required by this cohort of patients have not yet been quantified in the literature and this study addresses this issue.

## Methods

We conducted a retrospective audit examining the experience of a Specialist Tertiary Centre in the North West of England, with 8 years experience of performing this procedure, over an 18 month period (January 2009-June 2010). During this period 69 patients underwent cytoreductive surgery and HIPEC.

All patients received continuous thoracic epidural analgesia with 0.125% bupivocaine and 2 mcg/ml fentanyl. Regimes for induction and maintenance of anaesthesia were not standardized but determined by each individual anaesthetist.

Patients underwent a series of peritonectomy procedures to achieve complete cytoreduction. Peritoneal disease burden was assessed intraoperatively using the Peritoneal Cancer Index (PCI),[12] which scores 13 intra-abdominal sites from 0 (no disease) to 3 (lesion size > 5 cm) giving a possible range of scores from 0 to 39. HIPEC was only undertaken in patients in whom complete cytoreduction was achieved (a completeness of cytoreduction score < 1) [13].

After cytoreduction, the abdominal cavity was filled with 1.5% dextrose peritoneal dialysis solution (1.5-3 litres), then perfused at a temperature of 41.0°C to 42.5°C for 90 minutes. HIPEC was administered using either high dose mitomycin C (35 mg/m^2 ^in three pulses) for tumours of colorectal and appendiceal origin or doxorubicin (15 mg/m^2^) and cisplatinum (50 mg/m^2^) for peritoneal malignant mesothelioma.

Intra-operative fluids were guided by esophageal Doppler monitoring and the patient's hemodynamic status. All patients were extubated in the operating theatre and transferred to the Critical Care Unit (CCU) for initial post-operative management. Nutritional support with total parenteral nutrition (TPN) was commenced routinely on arrival to CCU converting to nasojejunal enteral nutrition after 2-3 days. Patient controlled epidural analgesia (PCEA), intravenous proton pump inhibitor (PPI) prophylaxis, nasogastric suction and low molecular weight heparin for prevention of venous thrombosis were given to all patients. Determination for the requirements of organ support and further interventions were made by the treating intensivist.

Analysis is based on data was gathered retrospectively from the hospital's peritoneal tumour service database and digital CCU notes (MetaVision^® ^iMDsoft, Neuss, Germany).

## Results and discussion

The average age of the patients in this study was 53.3 years (30-73) with 45 being female and 24 male. There was a mean Apache II score of 13.6 ranging from 2 to 26.

The appendix was the most common site of the primary tumour (see table 1). The mean length of surgery was 8.75 hours, including the administration of HIPEC, with an observed mean PCI score of 10.5. Table 2 shows the operative procedures performed.

**Table 1:** Primary site of tumour

Appendix	52
Colon	6
Caecum	3
Rectum	2
Ovary	2
Primary Peritoneal	1
Other/Unknown	3

**Table 2:** Operative procedures performed

Surgical procedure	Number performed
**Appendicectomy**	15
**Total greater omentectomy**	64
**Lesser omentectomy**	62
**Cholecystectomy**	40
**Splenectomy**	22
**Right colonic resection**	9
**Left colonic resection**	7
**Small bowel resection**	6
**Liver surface ablation**	24
**Right hemidiaphragm peritonectomy or ablation**	22
**Left hemidiaphragm peritonectomy or ablation**	12
**Total abdominal hysterectomy**	9
**Bilateral oophorectomy**	13
**Unilateral oophorectomy**	3
**Partial hepatectomy**	2
**Nephrectomy**	1
**Distal pancreatectomy**	1
**Orchidectomy**	1
**Partial cystectomy**	1

Patients needed to remain on the Critical Care Unit for 2.4 days (range 0.8-7.8 days) and length of hospital stay was 13 days (8-36 days) (see table 3). There were no 30 day mortalities.

**Table 3:** Details regarding surgery and length of stays

Peritoneal Cancer Index (PCI) score	10.5 (0-33)
**Length of surgery including HIPEC (hours)**	8.75 (6.4-12.6)
**Length of Critical Care Unit stay (days)**	2.4 (0.8-7.8)
**Length of Hospital stay (days)**	13 (8-36)

Despite the physiological changes that occur in patients undergoing cytoreductive surgery and HIPEC, the majority of patients (70.1%) did not require post-operative organ support. (see table 4). All patients were extubated immediately post-operatively in theatre and no patient required early re-intubation. 2 (3%) patients subsequently required respiratory support due to the development of post-operative pneumonia.

**Table 4:** Organ support interventions required post-operatively

Intervention	Number of patients
**Vasopressor support (Norepinephrine)**	18 (26.1%)
**Non-invasive ventilation**	1 (1.5%)
**Invasive Positive Pressure Ventilation**	1 (1.5%)
**Renal Replacement Therapy**	0

18 (26.1%) patients required vasopressor support with norepinephrine. The mean duration of vasopressor requirement was 13.94 hours (range 5-51 hours) with a mean dose of 0.04 mcg/kg/min. Predictably, the patients who had higher vasopressor requirements were those who had significant post-operative complications. No patients developed acute renal failure post-operatively.

Table 5 shows serial mean values of key blood parameters demonstrating the effects of cytoreductive surgery and HIPEC on clotting (INR and aPTT), platelets, haemoglobin and serum albumin and creatinine. There is a trend towards coagulopathy peaking 24 hours post-operatively although correction of abnormal coagulation with Fresh Frozen Plasma was only indicated in 1 patient. 15 patients required transfusion with Red Blood Cells in the post-operative period on CCU.

**Table 5:** Mean values of key laboratory tests

	Pre-operative	Immediately post-op	Day 1 post-op	Day 3 post-op
**Albumin (g/L)**	44.2	22.7*	23.6*	28.2*
**Creatinine (μmol/L)**	88.3	82.5*	77.9*	68.6*
**INR**	1.10	1.34*	1.47*	1.24*
**aPTT (secs)**	29.6	31.0*	35.5*	33.2*
**Platelets (x10^9^/L)**	306	238*	233*	219*
**Haemoglobin (g/dL)**	13.2	11.8*	10.7*	10.4*

(*) p < 0.05

There was a significant drop observed in serum albumin. All patients received immediate nutritional support with TPN on their arrival to the CCU. Renal function, reflected by no significant alterations in serum creatinine, was well preserved.

**Figure 1:**
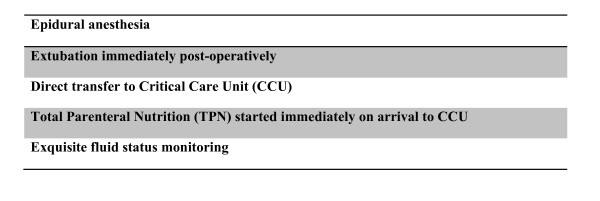
**A checklist for patients undergoing CRS and HIPEC**.

There was a low incidence of significant post-operative complications that necessitated interventions (see table 6). As described above, the 2 patients who developed pneumonia post-operatively required respiratory support via invasive positive pressure ventilation (IPPV) and non-invasive ventilation (NIV) respectively. One patient required the use of an intravenous glycerol-tri-nitrate (GTN) infusion for 4 days for uncontrolled hypertension. There were no surgical complications that necessitated re-operation and no haematological toxicity reactions to the HIPEC. Based upon our experience we have created a checklist of the key peri-operative interventions for patients undergoing CRS and HIPEC (see Figure 1).

**Table 6:** Significant complications occurring the post-operative period

COMPLICATION	NUMBER OF PATIENTS
**Pneumonia**	2 (2.9%)
**Line sepsis**	1 (1.5%)
**Uncontrolled hypertension**	1 (1.5%)
**Arrhythmias**	0
**Acute Renal Failure**	0
**Surgical complications, such as intestinal fistula, requiring further surgery**	0

Cytoreductive surgery and HIPEC results in a number of physiological changes with effects on the cardiovascular system, oxygen consumption and coagulation. Thus, there are many potential challenges presented to Critical Care Physicians in the immediate post-operative management.

There is an increase in airway pressure and a reduction in functional residual capacity intra-operatively as the administration of HIPEC causes elevation of the diaphragm as a result of increased intra-abdominal pressure [9]. However, all of our patients were extubated immediately post-operatively in the operating theatre with no complications and there is no evidence in our data to suggest any significant increase in post-operative oxygen requirements as a direct result of HIPEC administration. Schmitz et al. described 60% of their post-operative HIPEC patients being transferred to the ICU ventilated with a median ventilation of 3.7 hours although this period was 10.3 hours in patients without epidural analgesia [10]. With the aid of epidural analgesia our experience is that early extubation is safe conferring the benefits of reduced periods of ventilation.

In addition to the benefits of avoiding early post-operative ventilation, epidural analgesia is also associated with improved patient satisfaction [14]. Furthermore, it reduces the incidence of complications related to high dose intravenous opioids [15] and, in our experience, is valuable in aiding the early mobilization of patients.

There are significant amounts of fluid loss (up to 4 litres a day) in the first few days post CRS and HIPEC [11] and adequate fluid resuscitation to ensure end-organ perfusion is essential to prevent complications such as renal failure. The administration of HIPEC increases cardiac output and heart rate intra-operatively as a result of an increased metabolic rate [10]. Vasopressor support may be required for short periods particularly in the presence of thoracic epidural analgesia, but our data suggest that HIPEC has minimal cardiovascular sequelae in the post-operative period. This surgery is associated with significant protein losses perioperatively [16] which, alongside albumin diffusion into extravascular spaces as a consequence of intra-operative cytokine release, result in a marked drop in serum albumin and early administration of parenteral nutrition is important.

Coagulopathy is a recognized complication of this surgery [11]. It is probably dilutional in origin [17] due to the high volume of fluid resuscitation and shift, although there may be a direct effect from the HIPEC itself. Both our own experience and that of Schimdt et al. is that restoration of normal coagulation appears to have occurred within 72 hours [10].

Sepsis is the leading cause of mortality post-operatively and the nature of the procedure makes the patients at particularly high risk [18]. Haematological toxicity is a recognized complication of HIPEC and has been reported in up to 9% of patients [19]. Early recognition of post-operative complications, such as anastomotic leaks, intra-abdominal bleeding and abscesses, is a key aspect of Critical Care management.

Our centre has 8 years experience of performing cytoreductive surgery and HIPEC. The low incidence of need for organ support and other significant Critical Care interventions is in part likely due to improved and expert surgical technique which is associated with reductions in morbidity and mortality [5,7]. Other studies have reported peri-operative mortality rates of 3%[20] and 5% [6]. Furthermore, our patients had a shorter mean length of total hospital stay (13 days) than many centres (up to 29 days [7]) reflecting low complication rates but may also in part be due to longer periods of initial Critical Care monitoring.

The current cohort of patients undergoing this procedure is relatively young with few co-morbidities. In future, as the indications for HIPEC increase,[5,21] the patient population may be older with significant co-morbidities and their ability to tolerate the physiological stresses would need to be re-evaluated.

## Conclusion

Given the well described physiological abnormalities that occur during cytoreductive surgery and HIPEC the degree of organ support required is minimal. Early extubation is efficacious with the aid of epidural analgesia. Critical Care monitoring for 48 hours is still desirable in view of the challenges of fluid management, low albumin state, coagulopathy and potential complications.

## Conflict of interests

The authors declare that they have no competing interests.

## Authors' contributions

TC participated in the design of the study, data collection, statistical analysis and drafted the manuscript. PHM participated in the design of the study, statistical analysis, overseeing the project and revising the manuscript. Both authors read and approved the final manuscript.
